# Enhancing Phytoextraction Potential of *Brassica napus* for Contaminated Dredged Sediment Using Nitrogen Fertilizers and Organic Acids

**DOI:** 10.3390/plants13060818

**Published:** 2024-03-13

**Authors:** Nadežda Stojanov, Snežana Maletić, Jelena Beljin, Nina Đukanović, Biljana Kiprovski, Tijana Zeremski

**Affiliations:** 1 Institute of Field and Vegetable Crops, Maksima Gorkog 30, 21000 Novi Sad, Serbia; biljana.kiprovski@ifvcns.ns.ac.rs (B.K.); tijana.zeremski@ifvcns.ns.ac.rs (T.Z.); 2 Department of Chemistry, Biochemistry and Environmental Protection, Faculty of Sciences, University of Novi Sad, Trg Dositeja Obradovića 3, 21000 Novi Sad, Serbia; snezana.maletic@dh.uns.ac.rs (S.M.); jelena.beljin@dh.uns.ac.rs (J.B.); nina.djukanovic@dh.uns.ac.rs (N.Đ.)

**Keywords:** dredged sediment, heavy metals, *Brassica napus*, phytoremediation, nitrogen fertilizers, organic acids

## Abstract

Dredged sediment contaminated with heavy metals can be remediated through phytoremediation. The main challenge in phytoremediation is the limited availability of heavy metals for plant uptake, particularly in multi-contaminated soil or sediment. This study aimed to assess the effect of the nitrogen fertilizers (ammonium nitrate (AN), ammonium sulfate (AS), and urea (UR)), organic acids (oxalic (OA) and malic (MA) acids), and their combined addition to sediment on enhancing the bioavailability and phytoremediation efficiency of heavy metals. The sediment dredged from Begej Canal (Serbia) had high levels of Cr, Cd, Cu, and Pb and was used in pot experiments to cultivate energy crop rapeseed (*Brassica napus*), which is known for its tolerance to heavy metals. The highest accumulation and translocation of Cu, Cd, and Pb were observed in the treatment with AN at a dose of 150 mg N/kg (AN_150_), in which shoot biomass was also the highest. The application of OA and MA increased heavy metal uptake but resulted in the lowest biomass production. A combination of MA with N fertilizers showed high uptake and accumulation of Cr and Cu.

## 1. Introduction

Plants from the *Brassica* genus are extensively employed in phytoremediation due to their efficiency in terms of heavy metal uptake [1,2]. Rapeseed (*Brassica napus* L.) is known to be efficient in the uptake of various heavy metals, especially cadmium [3]. In addition to being cultivated as a crop for human and animal consumption, oil, and biodiesel production [4], *Brassica napus* could be used for phytoremediation. Moreover, the biomass of *Brassica napus* holds significant potential for the production of green energy [5]. One way of using such biomass is conversion in biorefineries. By utilizing Brassica napus harvest residues in that way, the soil would be cleaned, its functions improved, and biomass would be used for biofuel production without indirectly changing land use.

Heavy metals are non-biodegradable and can be taken up and accumulated in living organisms when present in a bioavailable form in the environment. In this way, they have the potential to transfer to higher trophic levels and cause harmful effects [6]. This environmental concern is emphasized by the global prevalence of sediment contamination with heavy metals recorded in Europe [7,8,9], China [10], the USA [11], and India [12]. More than 14 billion m^3^ of sediment in China [13] and around 300 million m^3^ in the USA [14] are dredged annually. According to the European network SedNet, up to 200 million m^3^ of sediment is dredged every year in Europe. Sediment is dredged to maintain the navigability of waterways, control floods, or remove contaminated sediment. Several potential usages of dredged sediment include its use in preventing coastal erosion, reconstructing landscapes [15], and usage as a construction and building material [16]. Additionally, there is potential for using dredged sediment in agriculture due to its high content of organic matter and other nutrients [17].

Addressing this concern, phytoremediation is one of the greenest and most cost-effective remediation techniques that could be applied. However, a significant challenge in this approach is the limited bioavailability of heavy metals. To improve metal uptake by plants, the potential application of chemically assisted phytoextraction using chelating agents from the group of amino polycarboxylic acids (EDTA, EDDS, and DTPA) has been investigated over the years. Although they are suggested to be efficient in enhancing metal uptake [3,18,19,20], there is the risk of heavy metal leaching due to the increased mobility of metal–chelator complexes [21], which are very stable [22]. Moreover, the half-life of EDTA in soil is reported to be more than 60 days [23].

In order to reduce the risk of leaching, as in cases when EDTA is used, some organic acids with significantly shorter half-lives were considered chelating agents [20,24]. Additionally, they could increase heavy metal mobility through acidifying soil or sediment and, most importantly, are easily degradable [25]. Citric acid has been mostly used in the phytoremediation of soils contaminated with heavy metals until now [26,27]. However, some organic acids that also have potential as chelating agents for phytoremediation enhancement are oxalic, acetic, malic, tartaric acid, and many others [20,24,28,29].

Additionally, phytoremediation could be enhanced with different soil amendments, such as sulfur [30], biochar [31], plant growth-promoting bacteria (PGPR) [32,33], and different fertilizers [34,35,36]. Nitrogen is an essential element for plant growth, and it is observed that it has beneficial effects not only on biomass production but also on heavy metal uptake in the phytoremediation process [37,38].

The aim of this study was to evaluate the effect of sediment amendments, including organic acids (oxalic and malic) and three N fertilizers (ammonium nitrate, ammonium sulfate, and urea), on the efficiency of phytoextraction in comparison to unamended sediment. Additionally, the synergistic effect of organic acids and N fertilizers on phytoremediation enhancement was compared to individual treatments, and the control was assessed. To our knowledge, the synergistic effect of organic acids and fertilizers on the improvement of phytoremediation has not been investigated so far. The contamination of the sediment used in this study was not simulated with the spiking of heavy metals prior to the experiment, but it was inherently multi-contaminated. Up to this point, there have been a few investigations into the potential enhancement of phytoremediation techniques applied to dredged sediment. The application of amendments that could enhance phytoremediation and make the sediment suitable for bioenergy production was assessed. To determine phytoremediation efficiency both with and without amendments, biomass production, oxidation stress parameters, bioconcentration (BCF), and translocation (TF) factors were determined and compared.

## 2. Results and Discussion

### 2.1. Sediment Physical and Chemical Properties

In order to characterize the sediment, the physical and chemical properties were determined (Table 1). Although there are Maximum Permissible Concentrations (MPCs) for heavy metals in sediment [39], this study focuses on target concentrations in soil because dredged sediment deposited in a landfill is used, and its potential agricultural application in the cultivation of energy crops was assessed. The total content of Cd, Cr, Cu, and Pb in the sediment exceeded the target values for metals in soil according to National Legislation (RS 50/2012), Dutch regulatory standards (2000), and soil and groundwater intervention values, which are 0.8 mg/kg for Cd, 100 mg/kg for Cr, 36 mg/kg for Cu, and 85 mg/kg for Pb. The concentrations of Ni and Zn were also higher than the target values, but in our experiments, the uptake of these elements by *Brassica napus* was generally low and not influenced by selected treatments, and for that reason, it is not presented in the paper. The distribution of heavy metals in the fractions according to the BCR procedure (Table 2) showed that Cr and Cu are found mainly in F3, bound to organic matter and sulfides. The oxidizable fraction (F3) is relatively stable, and the metals within it can become mobilized only under oxidizing conditions, resulting from organic matter degradation [40]. The metals present in F1 are considered the most mobile and readily available to plants, followed by F2 [40,41]. In our study, Cd was found mainly in F2, bound to Fe and Mn oxides with 50.9% of the total amount, and in F1 with 16.1%. In other studies, it is also found mostly in F1 and F2 and is considered a very mobile metal [42,43,44]. Lead, Pb, a heavy metal with low mobility, is mainly found in F3 bound to organic matter and in F4 bound to silicates. The metals in F4 are found inside the mineral crystal structure, which makes them less mobile and unavailable for plants [40].

**Figure 1:**
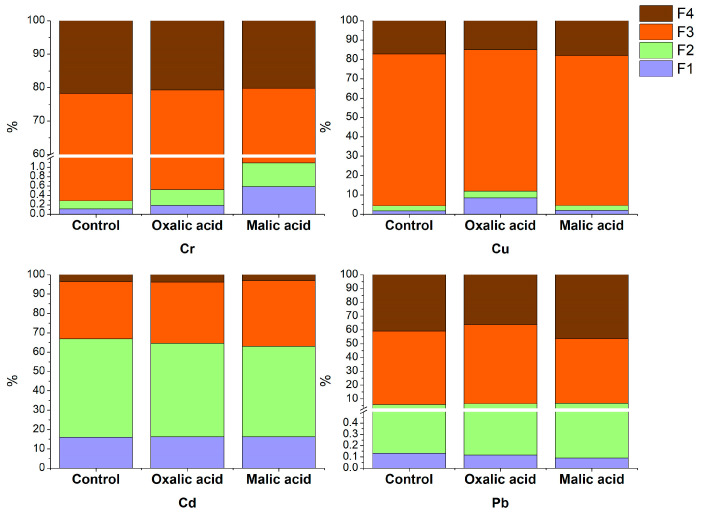
The percentage of heavy metals (Cr, Cu, Cd, and Pb) per BCR fraction in the control and after the addition of oxalic and malic acid.

The addition of oxalic acid increased the concentration of Cr in F1 and F2 by 81.1% and 105.9%, respectively, while the addition of malic acid increased the Cr concentration in F1 and F2 by 480.6% and 219.2%, respectively (Figure 1). The stability constant of Cr(III) with oxalic acid is 5.34 [45], and with malic acid, it is 5.4 [46]. Although the stability constant of the Cr complex with malic acid is only slightly higher, in this study, the concentration of chromium released into F1 is three times higher than that with oxalic acid. According to the results of other authors, the amount of Cr released is higher with malic acid compared to oxalic acid [47]. In addition, some studies reported that acids with more carboxylic and/or hydroxylic groups have more binding sites with negative charges, thus leading to higher Cr release [48]. With the addition of oxalic acid, the concentration of Cu in F1 and F2 increased by 519.1% and 47.1%, respectively, while malic acid increased the Cu concentration in F1 by 24.5%. It may be due to the stability constants of these organic acids with Cu, which are 4.84 with oxalic acid [49] and 3.60 with malic acid [50]. Neither malic nor oxalic acid significantly changed the distribution of Cd and Pb in the sediment fractions, nor did it increase the concentration of these heavy metals in F1 or F2 (Figure 1).

### 2.2. Influence of Nitrogen Fertilizers and Organic Acids Application on Biomass Production

In metal- and multi-contaminated soils, plants are grown under stress. A decrease in biomass is one of the first signs of the impact of stress on plants. In this study, the effect of sediment treatment with nitrogen fertilizers, organic acids, and combinations of fertilizers and organic acids on the rapeseed biomass was assessed. The dry matter of *Brassica napus* shoot and root grown under different treatments is presented in Table 3.

While the statistical analysis did not show a significant difference in shoot biomass between the treated plants and the control, it is observed that the application of lower doses of N fertilizers (150 mg N/kg sediment) increased shoot biomass. By contrast, plants grown in sediment with the addition of 300 mg N/kg had a lower shoot biomass than those grown with a lower dose of fertilizers, probably due to the phytotoxic effect of high nitrogen concentrations [51]. Applying organic acids to the sediment reduced shoot biomass, ranging from 32.2% with OA treatment to 50.0% with MA treatment compared to the control.

Interestingly, in sediment treatments with organic acids combined with N fertilizers, except for the OA+AN treatment, the shoot biomass was higher than in the treatments with the respective acid alone. This could suggest that nitrogen may mitigate the stress caused by heavy metals in plants. The mechanism of nitrogen and heavy metal interactions in soil has yet to be fully understood. However, nitrogen increases photosynthetic activity [38], and being an essential component of enzymes and proteins, it promotes plant growth and development. In our study, the highest increase in shoot biomass compared to the control was observed in the treatment with ammonium nitrate (AN_150_), which was 162.1% of the control shoot biomass. The reason for the high AN efficiency compared to other nitrogen fertilizers could be that nitrogen in this type of fertilizer originates from two forms of N easily accessible to plants (NH_4_^+^ and NO_3_^−^), which results in higher N uptake and utilization [52]. The root biomass of *Brassica napus* follows the same trend as the shoot biomass.

### 2.3. Influence of Nitrogen Fertilizer Application on Heavy Metal Uptake

During the nitrification process in soil, N fertilizers can decrease pH in the rhizosphere by releasing H^+^ ions [53] and, therefore, can increase metal mobility and bioavailability [37,54]. In our study, this effect was not observed, and the pH value of sediment remained unchanged after fertilizer addition.

**Figure 2:**
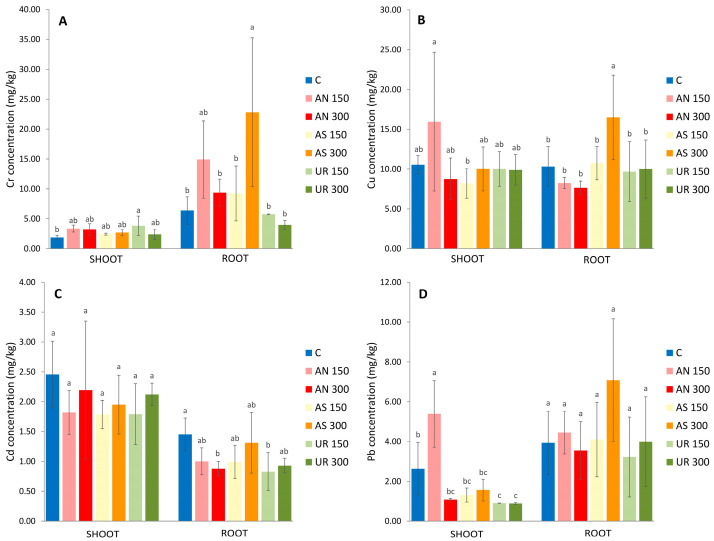
Mean concentration of (**A**): Cr, (**B**): Cu, (**C**): Cd, and (**D**): Pb in *Brassica napus* shoots and roots grown in sediment amended with ammonium nitrate (AN_150_ and AN_300_), ammonium sulfate (AS_150_ and AS_300_), and urea (UR_150_ and UR_300_) in the rate of 150 mg N/kg and 300 mg N/kg. Error bars represent standard deviation (*n* = 3), and different letters stand for significant difference between treatments (*p* < 0.05).

The concentration of Cr in *Brassica napus* shoots increased when nitrogen fertilizers were applied. However, the increase was significantly higher than the control only in the UR_150_ treatment, in which the Cr concentration was 3.83 mg/kg, two times higher than the control. At the same time, the concentration of Cr in roots was significantly increased only in the AS_300_ treatment, where the concentration of Cr was 22.79 mg/kg or 3.5 times higher than in the control roots (Figure 2A). Although applying N fertilizers increased Cd uptake by the shoots and roots of *Brassica napus*, its concentration remained very low compared to the Cr concentration in sediment (191.97 mg/kg) (Table 1).

The concentration of Cu in roots was significantly higher compared to the control in the AS_300_ treatment, reaching 16.50 mg/kg, which was about 1.6 times higher than the Cu concentration observed in the control roots (10.32 mg/kg). There was no significant difference in the Cu concentration in *Brassica napus* shoots in treatments with N fertilizers compared to the control (Figure 2B).

The concentration of Cd was highest when no fertilizers were applied, reaching 2.46 mg/kg in shoots and 1.45 mg/kg in roots (Figure 2C). No enhancement in the Cd concentration was observed in the presence of nitrogen fertilizers, either by shoots or roots. Similar to our results, *Kasiuliene* et al. (2016) [55] found no increase in Cd concentration in the shoots or roots of *Brassica napus* when urea was applied. Although some authors observed an increase in the Cd concentration in *Brassica napus* after applying N fertilizers [37], this could be because they used soil spiked with Cd, and the concentration of Cd in F1 was twice as high as in our study. The addition of ammonium sulfate and urea (200 mg N/kg soil) did not enhance Cd uptake by *Solanum nigrum* [56], which could suggest that the effect of the same nitrogen fertilizers depends on plant type and soil properties.

The accumulation of Pb in *Brassica napus* shoots and roots is shown in Figure 2D. The concentration of Pb in shoots in almost all treatments was lower than in the control (2.64 mg/kg). Nitrogen is transported from the roots to the shoot through the xylem, the same as Pb [57]. Therefore, the addition of nitrogen fertilizers could prevent Pb uptake and transport through the plant. Only in the AN_150_ treatment was the Pb concentration in shoots significantly higher than in the control, and it was 5.39 mg/kg. It is observed by Gul et al. (2019) [58] that ammonium nitrate could increase Pb accumulation in *Pelagronium hortroum*. Interestingly, the Pb concentration in shoots in the treatment with a higher dose of ammonium nitrate (AN_300_) was more than two times lower compared to the AN_150_ treatment. However, the total concentration of Pb in sediment was 162.09 mg/kg. Therefore, even the highest concentration measured in *Brassica napus* shoots was 26 times lower than in the sediment. Other authors also recorded low Pb uptake by *Brassica napus* [59,60,61,62].

### 2.4. Influence of Organic Acids and Combination of Organic Acids and Nitrogen Fertilizers on Heavy Metals Uptake by Brassica napus

Oxalic and malic acids act as chelating agents, thus increasing the solubility and bioavailability of metals by forming soluble metal–chelate complexes. The half-life of organic acids in soil usually depends on pH, soil type, and environmental conditions. The half-life of oxalic and malic acid is up to 4.5 days and shorter as soil layers are closer to the surface [25,63]. At this point, the effect of the addition of organic acids on the uptake of Cd [4,64], Ni [65], and Cu [66] by *Brassica napus* has been investigated. However, in these studies, the soil was not contaminated but amended with soluble salts of the investigated heavy metals. The effect of oxalic and malic acid on the uptake of heavy metals from multi-contaminated sediment by *Brassica napus* was not evaluated.

**Figure 3:**
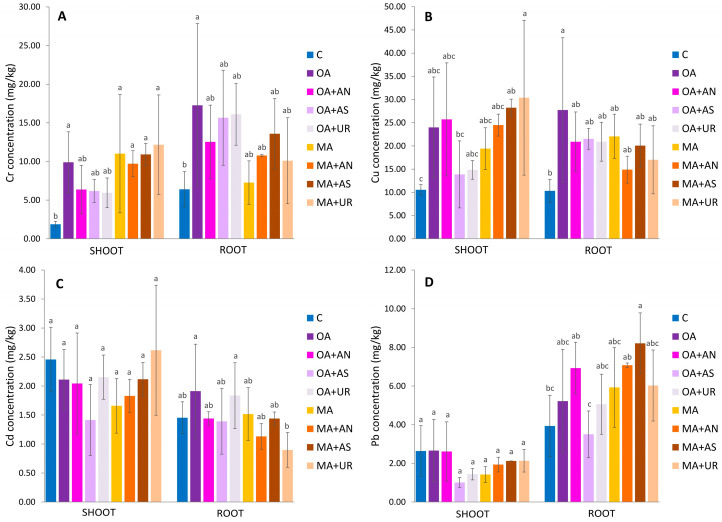
Mean concentration of (**A**): Cr, (**B**): Cu, (**C**): Cd, and (**D**): Pb in *Brassica napus* shoots and roots grown in sediment treated with 20 mmol/kg oxalic (OA) and malic acid (MA) and their combination with ammonium nitrate (AN), ammonium sulfate (AS), and urea (UR) in the rate of 150 mg N/kg. Error bars represent standard deviation (*n* = 3) and different letters stand for significant difference between treatments (*p* < 0.05).

The combined application of organic acids and nitrogen fertilizers was used to investigate their potential synergistic effect. The time of application of oxalic and malic acids and fertilizers is given in Section 3.1. The accumulation of Cr, Cu, Cd, and Pb is presented in Figure 3. 

**Figure 4:**
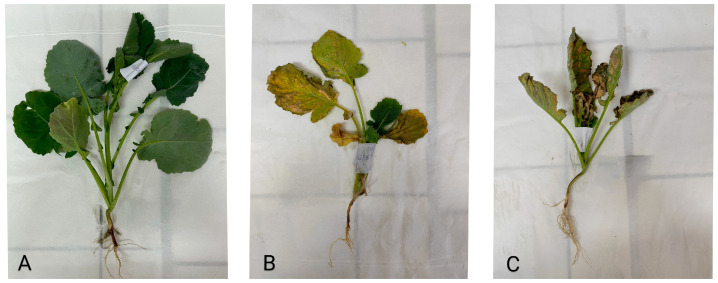
Photos of *Brassica napus*: (**A**) control plant, (**B**) plant under the OA treatment, (**C**) plant under the MA treatment.

The addition of oxalic and malic acid resulted in phytotoxic symptoms, as illustrated in Figure 4.

After sediment treatment with oxalic acid, the Cr concentration significantly increased in the shoots (9.90 mg/kg) and roots (17.27 mg/kg) compared to the control, in which the concentration of Cr in shoots and roots was 1.87 mg/kg and 6.40 mg/kg, respectively (Figure 3A). The addition of malic acid in the sediment caused a significant increase in the Cr concentration by *Brassica napus* shoots (11.01 mg/kg), which was almost six times higher than in the control. At the same time, the increase in roots was minor, recording 7.26 mg/kg compared to its concentration in control roots. Although malic acid released more Cr in F1 (Figure 1), the concentration of Cr in roots was more than two times higher when oxalic acid was applied. All treatments containing a combination of malic acids and nitrogen fertilizers enhanced the Cr concentration in *Brassica napus* shoots significantly compared to the control. The shoot concentration was 5.1, 5.8, and 6.4 times higher in the MA+AN, MA+AS, and MA+UR treatments than the Cr concentration in the control shoot, respectively. However, the concentration of Cr in *Brassica napus* was low compared to its concentration in sediment (Table 1), following other authors who found that Cr concentration in shoots of *Brassica napus* and *Brassica juncea* is significantly lower than in soil [33,67,68]. It suggests that *Brassica napus* possesses certain mechanisms to counteract the uptake of Cr, such as exclusion. Chromium is not regarded as an essential element for plants and tends to bind to the cell wall; thus, it is not translocated to upper plant parts [69].

With the addition of oxalic acid, the concentration of Cu was 23.99 mg/kg in *Brassica napus* shoots and 27.74 mg/kg in roots, 2.3 and 2.7 times higher than the control, respectively (Figure 3B). The addition of malic acid led to a 1.8 times higher concentration of Cu in shoots (19.42 mg/kg) and a double increase in the root concentration (22.05 mg/kg), both relative to the control. The higher concentrations of Cu in *Brassica napus* shoots and roots in the presence of oxalic acid are in accordance with its higher concentration in F1 and F2 sediment fractions when the oxalic acid is applied compared to the malic acid. Considering the synergistic effect of organic acids and nitrogen fertilizers, plants in the MA+AS and MA+UR treatments showed significantly higher values for Cu concentration in shoots, which were 28.24 mg/kg and 30.39 mg/kg, respectively. The presence of fertilizers enhanced Cu uptake in shoots compared to the MA treatment only. The combined treatments with oxalic acid led to a decrease in Cu concentration in shoots and roots compared to the OA treatment only.

The concentration of Cd in *Brassica napus* shoots and roots was not significantly enhanced by the addition of oxalic and malic acid nor by their combination with nitrogen fertilizers (Figure 3C). Other authors found that oxalic and malic acid increase Cd uptake by *Brassica napus* shoots [24,28]. However, in our study, sediment was contaminated with Cd, and F1 contained 0.71 mg/kg Cd, as opposed to a study by Li et al. (2020) [28] in which 20 mg/kg Cd was spiked as CdCl_2_ to the soil, which could influence the bioavailability of Cd to be higher. The study conducted by Qiao et al. (2020) [24] was conducted in soil contaminated with Cd (4.81 mg/kg), but they used sandy loam with a low organic matter content, which could be a reason for higher Cd mobility and availability [70].

The application of oxalic and malic acid had no significant effect on the increase in Pb concentration in *Brassica napus* shoots or roots (Figure 3D). Considering combined treatments, the concentration of Pb in shoots was lower than in control, while root concentration ranged from 3.50 mg/kg (OA+AN) to 8.21 mg/kg (MA+AS). The latter showed a significant increase, with a concentration in the roots that was two times higher than in the control roots. It was observed that the concentration of Pb in all treatments was higher in roots than in shoots of *Brassica napus*.

### 2.5. Influence of Sediment Amendments on Oxidative Stress Parameters

The plant accumulates heavy metals through the root system, xylem, and phloem loading, transferring them to the shoot, detoxifying and sequestering heavy metals at the cellular level, mainly in the vacuole [71]. Heavy metal uptake could significantly affect plant metabolism, disrupting photosynthesis and respiration, enhancing the production of reactive oxygen species (ROS), and leading to enzymatic and non-enzymatic antioxidants for ion homeostasis [72,73].

**Figure 5:**
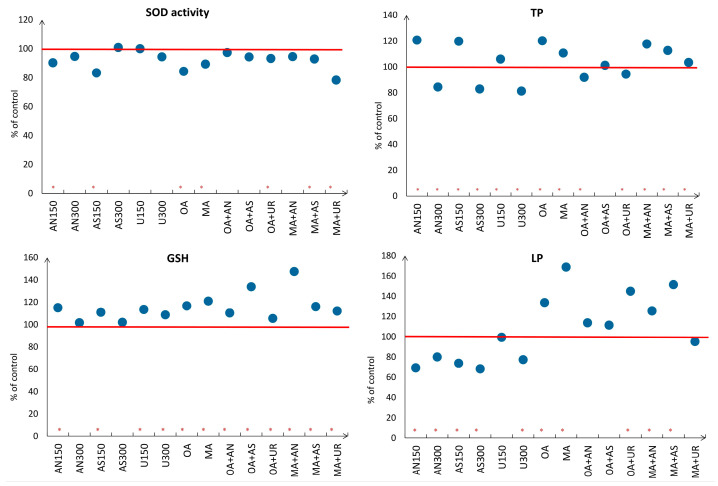
Change in oxidative stress parameters in comparison to control (100%, red line): lipid peroxidation intensity (LP), superoxide dismutase activity (SOD), total phenolics (TP), and reduced glutathione (GSH) content in leaves of rapeseed plants treated with respective treatments (abbreviations explained in Section 3.1). An asterisk indicates a significant difference between treatments and respective control.

The rapeseed plants used in this research treated with fertilizers had lower lipid peroxidation intensity than those from the control (1–1.5 times lower than the control value) and treatments with acids. The highest LP intensity had MA treatment (48.65 nmol/g), almost two times that of the control value, while plants in the UR_150_, OA+AN, OA+AS, and MA+UR treatments did not differ from the control (Figure 5). Chen et al. (2009) [74] tested the impact of OA applied on the leaves of six-week-old *Arabidopsis* seedlings (30 mmol/L OA) and recorded values from 80 to 600 nmol/g, which could serve as a reference range of values for LP intensity under OA stress. Zaheer et al. (2020) [75] measured 35 µmol/g of MDA content and 22 U/g SOD activity in the leaves of rapeseed plants exposed for 30 days to Zn and Cr-contaminated tannery wastewater. Lietão et al. (2021) [76] exposed 20 days-old rapeseed seedlings to 18 days of 100 µM Cu and 50 µM Cd and reported LP values of approx. 2310 and 1120 nmol/g, while control plants had an LP of 1960 nmol/g. The SOD activity of the tested plants was approx. 189, 211, and 189 U/g for the control plants and 100 µM Cu and 50 µM Cd treatments, respectively. Jahan et al. (2021) [77] recorded 55–130 U/g SOD activity in the leaves of 14 days-old rapeseeds grown for seven days on a substrate exposed to chromium. As for the current experiment, the values of SOD activities ranged from 174 to 240.6 U/g. The activity of SOD was significantly lower in the leaves of plants in the AN_150_, AS_150_, OA, MA, OA+UR, MA+AS, and MA+UR treatments compared to the control plants (212 U/g on average).

The content of total phenolics ranged from 0.9 to 1.9 mg/g in the fresh leaves, and there was a significant accumulation of these compounds in all of the treated plants in contrast to the control, except for the plants treated with OA+AS. In an experiment conducted on rapeseed seedlings grown for 8 days under hydroponic conditions (100 µM Cd solution), plants with a higher accumulation potential or tolerance to high Cd accumulation (280 µg Cd/g dry weight) synthesized higher amounts of total anthocyanins, while the sensitive, low-Cd-accumulating genotype (90 µg Cd/g dry weight) had a higher content of flavonones, flavones, chalcones, quinones, dihydroflavonols, and flavonols [72]. The total flavonoid content ranged from 0.3–1.8 to 0.65–1.2 mg/g of the fresh weight in low Cd and high Cd-accumulating genotypes, respectively.

The values of reduced glutathione ranged from 10 to 19.1 µM/g in the plants tested in this experiment. A significant difference in GSH content was recorded in all treatments compared to the control, except for the AN_300_ and AS_300_ treatments. Chen et al. (2009) [74] obtained similar values of GSH when analyzing the activity of OA applied on leaves of six-week-old *Arabidopsis* seedlings and recorded values from 3 to 12 µM/g throughout 24h after the treatment, while Jahan et al. (2021) [77] recorded 0.01–0.016 µmole/g in 14 days-old canola grown for seven days on a substrate exposed to hexavalent chromium.

### 2.6. Overall Efficiency of Nitrogen Fertilizers and Organic Acids in Enhancing Brassica napus Potential for Phytoextractions

Besides the concentration of metals in plant tissue, the parameters used to assess phytoextraction efficacy are bioconcentration (BCF), translocation factor (TF), and the mass of accumulated metals in above-ground plant parts. The values for BCF, TF, and accumulated metal mass in shoots for *Brassica napus* are presented in Table 4.

BCF shows the plant’s ability to uptake heavy metals from the soil. The BCF values for Pb were the lowest, and they ranged from 0.008 to 0.037, while the highest BCF values were calculated for Cd and ranged from 0.271 to 0.460. The BCF values obtained in our study are comparable to the values of other authors for Pb in *Brassica juncea*, ranging from 0.002 to 0.039 [78] and in *Brassica napus* (0.100) [79]. The average BCF values in decreasing order are for Cd > Cu > Cr > Pb, which correlates well with the highest abundance of Cd and Cu in F1. Although the treatments did not increase BCF for Cd, it showed the highest bioconcentration potential in this study, even in the control treatment. It could be because *Brassica napus* can tolerate high concentrations of cadmium, which is confirmed by other authors [24,64,80].

TF indicates the plant’s ability to relocate heavy metals from roots to above-ground tissue. Plants with a TF value higher than 1 are considered suitable for phytoextraction. Moreover, if plants are grown in multi-contaminated soil or sediment, it is favorable that TF is high for as many heavy metals as possible. Considering this, in our study, the AN_150_ treatment was the only one with TF > 1 for Cu, Cd, and Pb, and the MA+AN and MA+UR treatments were the only ones with TF > 1 for Cr, Cu, and Pb. The highest TF was for Cd, ranging from 1.023 to 2.916 (Table 4).

Besides metal concentration in shoot and root, plant potential for phytoextraction is defined by the total heavy metal mass accumulated in above-ground plant parts. This value is affected not only by heavy metal concentration but also by shoot biomass. The mass accumulated in the *Brassica napus* shoot is shown in Table 4. The highest accumulation of Cu, Cd, and Pb was obtained in the AN_150_ treatment, while the mass of Cr accumulated in this treatment was similar to that in the MA treatment. In the treatments with organic acids, the concentration of Cr and Cu accumulated in shoots was 5.3–5.9 and 1.8–2.3 times higher, respectively, compared to the masses of these metals accumulated in the control. However, the treatments with organic acids caused the symptoms of phytotoxicity in plants (Figure 4), which caused the shoot biomass in the OA and MA treatment to be lower than the control. Consequently, the mass of the accumulated metals in the shoot in OA and MA treatment was low.

**Figure 6:**
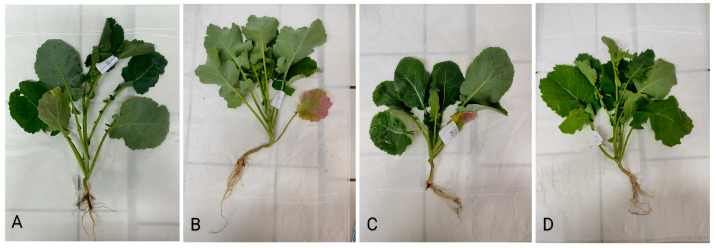
Photos of *Brassica napus*: (**A**) control plant, (**B**) plant under the AN_150_ treatment, (**C**) plant under the AS_150_ treatment, and (**D**) plant under the UR_150_ treatment.

In all treatments with nitrogen fertilizers, except UR_300_, the shoot biomass was higher than the control shoot biomass (Figure 6). However, only AN_150_ treatment resulted in a significantly increased accumulation of Cu and Pb in the *Brassica napus* shoot. The accumulation of heavy metals in all other treatments with nitrogen fertilizers was similar to or lower than that of the control. In other studies examining the influence of fertilizers on the uptake of heavy metals by plants, the observed increase in heavy metals uptake was attributed to a decrease in soil pH. The lower pH enhances the mobility and bioavailability of metals in the rhizosphere [37,81]. Since ammonium nitrate is a physiologically neutral fertilizer and has no significant effect on sediment pH, the increased uptake of Cu and Pb in our experiments cannot be explained that way. Interestingly, in our study, Cd uptake was not affected by treatment with fertilizers, although other authors found that an increased amount of N, regardless of its form, enhances Cd uptake, translocation, and accumulation in plants; moreover, nitrate promotes Cd uptake more than any other N form [37,56,58]. Our results showed that only ammonium nitrate in lower doses influenced the uptake of heavy metals, which indicates that more studies on the influence of fertilizer types and application rates, as well as mechanisms of interaction between heavy metals and nitrogen from fertilizers, are needed. Oxalic and malic acid were more selective in mobilizing metals than fertilizers. The uptake of Cr and Cu was increased in OA and MA treatment, while in the same treatments, the uptake of Cd and Pb remained similar to the control. This is because both acids form stable water-soluble complexes with Cr and Cu (Figure 1), increasing these metals’ bioavailability. However, the bioavailability of Cd and Pb did not change after adding organic acids.

Considering the stimulating effect of nitrogen fertilizers on plant biomass production, it could be assumed that their addition, in combination with organic acid, would lead to increased shoot biomass. Nonetheless, this effect was noticed only in MA treatments combined with fertilizers. When the OA treatments were combined with fertilizers, *Brassica napus* shoot biomass was comparable to shoot biomass in the OA treatment alone. Remarkably, combining the OA treatment with fertilizers resulted in a decrease in heavy metal uptake compared to the OA treatment alone. Conversely, the combination of MA with fertilizers led to an increase in heavy metal uptake compared to the MA treatment alone. The concentration of Cr and Cu in plants treated with MA and fertilizers was equal to or higher than their concentrations in the MA treatment without fertilizers. This effect was most noticeable in the MA+UR treatment, while the MA+AN treatment resulted in an increase in shoot biomass but not in the uptake of heavy metals compared to the MA treatment alone. The increased concentration of Cr and Cu and the increased shoot biomass resulted in the accumulated mass of Cr and Cu in the shoot of *Brassica napus* in the MA treatments combined with fertilizers being significantly higher than in other treatments. Since LP intensity was lower in the plants in the treatments combining organic acids and fertilizers than in the plants treated only with organic acids, it could be assumed that nitrogen fertilizers decrease oxidative stress caused by heavy metals. This suggests a synergistic effect of fertilizers and organic acids.

There are few studies investigating the phytoremediation potential of *Brassica napus* for multi-contaminated soils [3,61,79,82]. Most authors investigated the uptake of one or two metals spiked to the soil. Therefore, it is hardly comparable to our results. Our results for the accumulation of Cu in shoots were higher than those by Zaheer et al. (2015) [66]. According to them, the accumulation of Cu in *Brassica napus* shoots was lower than 3.5 µg even in the treatments with citric acid. In our study, the highest mass of Cd was 9.83 µg detected in the AN_150_ treatment. Other researchers recorded the mass of Cd in *Brassica napus* shoot in the range from 11.96 to 23 µg in the treatments with the addition of EDTA and citric acid [64] and in the range from 109.93 µg to 264.27 µg [37] with the application of N-fertilizers, which is higher than in our findings. However, in contrast to real multi-contaminated sediment used in our study, their studies involved soil spiked with Cd, which could be the reason for higher mobility and bioavailability, thus the uptake and accumulation of Cd. Shi et al. (2017) [60] found that the accumulation of Cd in *Brassica napus* shoots grown in soil contaminated with 3.25 mg/kg of Cd was lower than 5 µg, which is more similar to our results. There is not much data on Cr and Pb mass accumulated in *Brassica napus* shoots, but their concentration and accumulation remained low [60,67,68].

## 3. Materials and Methods

### 3.1. Pot Experiment Setup

Contaminated sediment used for pot experiments originated from the Begej Canal and was located at a disposal site in Srpski Itebej, Serbia, near the Serbian–Romanian border. The disposal site is located between the parallels 45°34′50.45″ N, 20°45′35.08″ E and 45°34′50.22″ N, 20°45′31.18″ E. The sediment was collected, manually mixed, and homogenized before being placed in pots. A representative sample of sediment was taken in order to characterize physicochemical properties and determine heavy metal content. For the experiment, Mitscherlich pots were filled with 5 kg of sediment each. The spring variety of rapeseed (*Brassica napus* L.) “Jovana” used for this study was provided by the Institute of Field and Vegetable Crops, Novi Sad, Serbia. Ten seeds of rapeseed were sown in the 2 cm top layer of the sediment in each pot and initially watered manually. Thirteen days after germination, two plants were maintained in each pot. Pot experiments were conducted in semi-controlled conditions, in an open-air space, protected from precipitation and equipped with an irrigation system. Pots were watered with deionized water to maintain constant sediment moisture at 2/3 of field capacity. The sediment was treated with three nitric fertilizers: ammonium nitrate (AN), ammonium sulfate (AS), and urea (UR), and two organic acids: oxalic (OA) and malic (MA), as explained in Table 5. All chemicals were purchased from Merck, Darmstadt, Germany. Nitric fertilizers were added 8 days after sowing. The amount of fertilizers in solid form for each pot was dissolved in 100 mL of distilled water and added to the pot. Subsequently, 100 mL of distilled water was added to the pot. The same procedure was applied for oxalic and malic acid, which were added 35 days after sowing.

Each treatment was conducted in 3 replicates. Both plants from each pot were sampled six weeks after the sowing. One plant from each pot was used for the determination of the oxidative stress parameters, and the other was used for the determination of the biomass and heavy metals content. Additionally, leaf samples were collected for the testing of the oxidative stress parameters and stored at −20 °C until the analysis. After sampling, the roots and shoots of each plant were separated and washed with distilled water. The shoots were rinsed using a laboratory bottle with approximately 200–300 mL of water. In order to remove sediment particles, the roots were submerged in a glass beaker filled with 300 mL of distilled water. It was repeated at least three times or until there were no visible sediment particles. Afterward, the shoots and roots were air-dried and ground using the Ika A10 Basic analytical mill (Ika, Staufen, Germany). Before grinding, the shoot height, shoot mass, and root mass of all plant samples were measured. The heavy metal contents in the shoots and roots were determined, respectively. After plant sampling, sediment was removed from the pots, and composites from all replicates of each treatment were made, air-dried, ground in a laboratory mill and used for subsequent physicochemical analysis and heavy metal content determination. In order to assess the influence of the addition of organic acids to sediment on heavy metal mobility, a laboratory experiment was performed. In this study, 10 g of sediment was mixed with 10 mL of oxalic or malic acid in a concentration that corresponded to 20 mmol of each acid per kg of sediment. After 24 h, the dry samples were homogenized, and the concentrations of heavy metals in the sediment fractions were determined in three replicates according to the BCR procedure described by Arain et al. [83].

### 3.2. Methods of Sediment and Plant Analysis

For the determination of the heavy metal contents in sediment samples, a mass of 1 g was mixed with 9 mL of ccHNO_3_ and 3 mL of ccHCl and digested at 170 °C in the microwave Start E/0610030141 (Milestone, Sorisole, Italy,) according to EPA 3051A [84]. After microwave digestion, the samples were filtered and diluted up to 50 mL with deionized water, and the heavy metal content was analyzed using the ICP-MS 7700 (Agilent Technologies, Tokio, Japan) according to EPA 6020B [85].

A mass of 0.5 g of plant samples was weighed and mixed with 7 mL of ccHNO_3_ and 2 mL of H_2_O_2_. The following temperature program was used for microwave digestion: heated to 85 °C for 4 min, 145 °C for 9 min, 200 °C for 4 min, and then kept at a 200 °C for 14 min. After microwave digestion, the samples were filtered and diluted up to 50 mL with deionized water, and the content was analyzed using ICP-MS (Agilent Technologies 7700) according to EPA 6020B.

The bioavailability of heavy metals was determined through the use of BCR sequential analysis, according to the procedure described by Arain et al. [83]. The number of heavy metals was determined in four fractions: acid-soluble (F1), reducible (F2), oxidizable (F3), and residual fraction (F4). After microwave digestion, the heavy metal content in each fraction was analyzed using ICP-MS (Agilent Technologies 7700) according to EPA 6020B.

The pH value of the sediment was determined using a glass electrode in a 1:5 (*w*/*w*) suspension of sediment and water, according to ISO 10390:2005 [86].

The available P and K content was determined via extraction with ammonium lactate solution, according to Egner and Riehm [87]. The available P content was analyzed using a UV/VIS spectrophotometer Cary 60 (Agilent Technologies, Waldbronn, Germany) at 830 nm, previously colored with ammonium molybdate, while the available K content was determined via flame photometry.

According to ISO 11277:2009 [88], the sediment texture determination samples were sieved through a series of sieves in the range of 2–0.063 mm. The sedimentation method was used for fraction 0.063 to <0.002 mm and by withdrawing the suspension at a defined time and depth in the cylinder.

The total N content was determined using the Kjeldal method (ISO 11261:1995 [89]) via the digestion of the sediment sample with ccH_2_SO_4_, distillation in H_3_BO_3_, and titration with HCl.

### 3.3. Determination of Oxidative Stress Parameters

Additional samples of rapeseed leaves were taken to investigate possible oxidative stress in the treated rapeseed plants. The intensity of lipid peroxidation (LP) was determined based on the content of malondialdehyde (MDA), one of the end products of membrane lipid breakdown in cells [90]. The activity of superoxide dismutase (SOD, EC 1.15.11) was determined according to the method [91] based on the principle of the ability of the SOD to inhibit the photochemical reduction of nitro blue tetrazolium chloride (NBT) with superoxide anion (O_2_^.−^). The content of reduced glutathione (GSH) was determined based on the color reaction of non-protein thiol (-SH) groups in the presence of 5,5-dithiobis-(2-nitrobenzoic acid) (DTNB) or Ellman’s reagent [92]. The content of total phenolics (TPs) was determined via a modified method described in Makkar (2003) [93]. All analyses were performed on a PerkinElmer UV/Vis Lambda 25 spectrophotometer (USA). Extractions were performed in two repetitions. The results represent the mean of two replicates.

### 3.4. Data Analysis

The mean and standard deviation of all measurements were determined. All of the data were statistically analyzed via one-way ANOVA following Duncun’s post test (*p* < 0.05) with Statistica 13.3 software for Windows. In order to evaluate phytoremediation efficiency, bioconcentration (BCF) and translocation (TF) factors were calculated.

The bioconcentration (BCF) factor shows the ability of the plant to uptake heavy metals from the sediment and is calculated (Equation (1)) as the ratio of the heavy metal concentration in the plant tissue (C*_plant_*) to the heavy metal concentration in the soil (C*_sediment_*) [20].

BCF = C*_plant_* (mg/kg)/C*_sediment_* (mg/kg) (1)

TF indicates the ability of the plant to relocate heavy metals from the roots to above-ground tissues (leaves, stems, seeds, etc.) and thus is calculated (Equation (2)) as a ratio of the heavy metal concentration in shoots (C*_shoots_*) to the concentration of heavy metals in the root (C*_roots_*) [20].

TF = C*_shoots_* (mg/kg)/C*_roots_* (mg/kg). (2)

## 4. Conclusions

Ammonium nitrate in a dose of 150 mg N/kg was found to be the most efficient for the uptake of Cr, Cu, and Pb by *Brassica napus* and for the translocation of these heavy metals. All of the heavy metals investigated (Cr, Cu, Cd, and Pb) showed high accumulation in above-ground plant parts when ammonium nitrate was applied. Organic acids (oxalic and malic) increased Cr and Cu concentration in an exchangeable sediment fraction, thus causing higher uptake of these heavy metals, but with the lowest biomass production and, therefore, the lowest absolute amount of metals. Although malic acid was less efficient regarding the uptake and accumulation of heavy metals, its combination with N fertilizers showed the highest uptake and accumulation of Cr and Cu, especially. Brassica napus, as an energy crop, has the potential for usage for phytoremediation of multi-contaminated sediment, with further investigation required through field experiments involving ammonium nitrate as the most potent N fertilizer for stimulating both heavy metals accumulation and biomass production.

## Figures and Tables

**Table 1:** Physicochemical properties of the sediment.

Parameter	Value
pH	7.19 ± 0.03
Conductivity (µS/cm)	940.6 ± 126.9
CEC (meg/100 g sample)	1.19 ± 0.1
Organic matter (%)	9.20 ± 0.47
Total N (%)	0.25 ± 0.02
Total S (%)	0.16 ± 0.02
Total P (g/kg)	1470.95 ± 116.81
Available P (mg/kg)	808.98 ± 284.63
Total K (mg/kg)	5517.1 ± 1148.1
Available K (mg/kg)	221.86 ± 22.59
Total Na (mg/kg)	523.7 ± 96.4
Total Mg (mg/kg)	8367.2 ± 1124.7
Total Ca (mg/kg)	528.5 ± 250.5
Total Cr (mg/kg)	191.97 ± 2.32
Total Ni (mg/kg)	39.56 ± 3.80
Total Cu (mg/kg)	131.98 ± 1.27
Total Zn (mg/kg)	515.83 ± 13.50
Total As (mg/kg)	0.89 ± 0.10
Total Cd (mg/kg)	4.42 ± 0.11
Total Pb (mg/kg)	162.09 ± 2.77
Sand % (50 µm–2000 µm)	38.7 ± 7.2
Silt % (2 µm–50 µm)	30.6 ± 10.9
Clay % (<2 µm)	30.6 ± 4.1

**Table 2:** The concentration of Cr, Cu, Cd, and Pb in mg/kg by BCR fractions in sediment.

	Water and Acid Soluble Fration F1 (mg/kg)	Reducible Fraction F2 (mg/kg)	Oxidizable Fraction F3 (mg/kg)	Residual Fraction F4 (mg/kg)
Cr	0.21 ± 0.00	0.33 ± 0.06	149.65 ± 4.53	41.77 ± 2.60
Cu	2.07 ± 0.10	3.66 ± 0.52	103.54 ± 3.87	22.71 ± 2.44
Cd	0.71 ± 0.05	2.25 ± 0.09	1.31 ± 0.13	0.16 ± 0.03
Pb	0.21 ± 0.02	9.10 ± 1.74	86.28 ± 16.61	66.49 ± 17.80

**Table 3:** Shoot and root biomass (dry matter) of *Brassica napus* under different treatments. Standard deviations are based on three replications, and different letters stand for significant differences between treatments (*p* < 0.05).

Treatment	Shoot Biomass (g)	Root Biomass (g)
C	3.33 ± 0.80 ^abcd^	0.54 ± 0.02 ^abcd^
AN_150_	5.40 ± 2.18 ^a^	0.79 ± 0.08 ^a^
AN_300_	4.39 ± 0.69 ^abc^	0.53 ± 0.18 ^abcd^
AS_150_	4.60 ± 1.65 ^abc^	0.70 ± 0.32 ^ab^
AS_300_	4.02 ± 0.85 ^abcd^	0.59 ± 0.13 ^abc^
UR_150_	4.82 ± 2.14 ^ab^	0.61 ± 0.18 ^abc^
UR_300_	3.26 ± 1.62 ^abcd^	0.46 ± 0.17 ^bcdefg^
OA	2.26 ± 1.39 ^cd^	0.23 ± 0.10 ^fg^
MA	1.67 ± 0.60 ^d^	0.20 ± 0.12 ^g^
OA+AN	1.86 ± 1.11 ^d^	0.28 ± 0.12 ^dfg^
OA+AS	2.75 ± 0.47 ^bcd^	0.36 ± 0.09 ^cdefg^
OA+UR	2.48 ± 0.73 ^bcd^	0.40 ± 0.03 ^cdefg^
MA+AN	3.23 ± 0.86 ^abcd^	0.48 ± 0.12 ^bcdef^
MA+AS	2.81 ± 1.01 ^bcd^	0.38 ± 0.12 ^cdefg^
MA+UR	3.07 ± 0.61 ^abcd^	0.45 ± 0.10 ^bcdefg^

**Table 4:** Bioconcentration (BCF) and translocation factor (TF) for *Brassica napus* metal uptake, and total mass of heavy metals accumulated in the *Brassica napus* shoot. Different letters stand for significant differences between treatments (*p* < 0.05).

Treatment	BCF				TF				Accumulated Mass in Shoot (µg)			
	Cr	Cu	Cd	Pb	Cr	Cu	Cd	Pb	Cr	Cu	Cd	Pb
C	0.011 ± 0.003 ^d^	0.078 ± 0.010 ^d^	0.445 ± 0.117 ^a^	0.020 ± 0.006 ^b^	0.319 ± 0.116 ^c^	1.049 ± 0.176 ^bcd^	1.683 ± 0.094 ^bc^	0.915 ± 0.911 ^ab^	6.23 ± 1.26 ^c^	35.13 ± 3.78 ^d^	8.19 ± 1.85 ^ab^	8.78 ± 4.38 ^b^
AN_150_	0.022 ± 0.002 ^cd^	0.111 ± 0.057 ^cd^	0.329 ± 0.075 ^a^	0.037 ± 0.008 ^a^	0.265 ± 0.147 ^c^	2.007 ± 1.285 ^a^	1.876 ± 0.525 ^bc^	1.192 ± 0.536 ^a^	18.17 ± 3.17 ^bc^	86.21 ± 47.08 ^ab^	9.83 ± 1.99 ^a^	80.31 ± 34.37 ^a^
AN_300_	0.018 ± 0.004 ^d^	0.064 ± 0.018 ^d^	0.394 ± 0.236 ^a^	0.010 ± 0.005 ^b^	0.359 ± 0.133 ^c^	1.130 ± 0.206 ^bcd^	2.421 ± 0.998 ^ab^	0.324 ± 0.125 ^bc^	14.09 ± 4.20 ^c^	38.37 ± 11.52 ^d^	9.62 ± 5.07 ^a^	14.78 ± 5.14 ^b^
AS_150_	0.015 ± 0.006 ^cd^	0.063 ± 0.010 ^d^	0.322 ± 0.039 ^a^	0.012 ± 0.002 ^b^	0.413 ± 0.078 ^c^	0.802 ± 0.296 ^cd^	1.923 ± 0.659 ^bc^	0.379 ± 0.195 ^bc^	18.57 ± 12.80 ^bc^	37.76 ± 8.52 ^d^	8.21 ± 1.09 ^ab^	6.03 ± 1.60 ^b^
AS_300_	0.024 ± 0.011 ^bcd^	0.080 ± 0.022 ^d^	0.359 ± 0.112 ^a^	0.016 ± 0.005 ^b^	0.134 ± 0.042 ^c^	0.636 ± 0.184 ^cd^	1.536 ± 0.182 ^bc^	0.230 ± 0.036 ^c^	10.83 ± 2.15 ^c^	40.27 ± 11.10 ^cd^	7.84 ± 1.98 ^abc^	6.28 ± 2.19 ^b^
UR_150_	0.018 ± 0.007 ^d^	0.074 ± 0.018 ^d^	0.323 ± 0.110 ^a^	0.008 ± 0.004 ^b^	0.508 ± 0.378 ^c^	1.085 ± 0.188 ^bcd^	2.211 ± 0.264 ^ab^	0.679 ± 0.617 ^abc^	18.45 ± 7.72 ^bc^	48.26 ± 10.54 ^bcd^	8.62 ± 2.45 ^ab^	8.12 ± 6.57 ^b^
UR_300_	0.012 ± 0.004 ^d^	0.073 ± 0.011 ^d^	0.379 ± 0.039 ^a^	0.009 ± 0.004 ^b^	0.392 ± 0.226 ^c^	1.146 ± 0.693 ^bcd^	2.305 ± 0.278 ^ab^	0.417 ± 0.223 ^bc^	7.82 ± 2.76 ^c^	32.28 ± 6.24 ^d^	6.93 ± 0.61 ^abcd^	4.61 ± 3.00 ^b^
OA	0.048 ± 0.024 ^ab^	0.180 ± 0.085 ^abc^	0.402 ± 0.124 ^a^	0.021 ± 0.011 ^b^	0.647 ± 0.195 ^c^	0.904 ± 0.133 ^bcd^	1.175 ± 0.261 ^c^	0.487 ± 0.067 ^bc^	22.35 ± 8.89 ^abc^	54.17 ± 24.53 ^bcd^	4.76 ± 1.18 ^bcde^	6.00 ± 3.65 ^b^
MA	0.048 ± 0.032 ^b^	0.186 ± 0.077 ^abc^	0.380 ± 0.179 ^a^	0.022 ± 0.008 ^b^	0.305 ± 0.183 ^c^	0.359 ± 0.093 ^d^	1.023 ± 0.829 ^c^	0.283 ± 0.265 ^bc^	18.35 ± 12.76 ^bc^	42.85 ± 20.29 ^cd^	3.40 ± 1.45 ^de^	4.35 ± 2.56 ^b^
OA+AN	0.032 ± 0.017 ^abcd^	0.110 ± 0.049 ^cd^	0.271 ± 0.136 ^a^	0.010 ± 0.002 ^b^	0.525 ± 0.187 ^c^	0.630 ± 0.305 ^cd^	1.024 ± 0.129 ^c^	0.319 ± 0.157 ^bc^	11.82 ± 5.82 ^c^	25.75 ± 13.42 ^d^	2.62 ± 1.14 ^e^	1.87 ± 0.47 ^b^
OA+AS	0.033 ± 0.009 ^abcd^	0.115 ± 0.012 ^bcd^	0.406 ± 0.085 ^a^	0.013 ± 0.001 ^b^	0.438 ± 0.191 ^c^	0.738 ± 0.238 ^cd^	1.221 ± 0.317 ^c^	0.315 ± 0.153 ^bc^	17.00 ± 4.10 ^bc^	40.83 ± 5.55 ^cd^	5.93 ± 1.05 ^abcde^	3.97 ± 0.82 ^b^
OA+UR	0.033 ± 0.006 ^abcd^	0.146 ± 0.024 ^abcd^	0.315 ± 0.095 ^a^	0.015 ± 0.001 ^b^	0.398 ± 0.185 ^c^	0.946 ± 0.453 ^bcd^	1.142 ± 0.422 ^c^	0.275 ± 0.152 ^bc^	14.75 ± 4.75 ^bc^	48.19 ± 11.18 ^bcd^	4.12 ± 1.17 ^cde^	3.53 ± 1.03 ^b^
MA+AN	0.045 ± 0.008 ^abc^	0.172 ± 0.013 ^abc^	0.334 ± 0.063 ^a^	0.019 ± 0.002 ^b^	1.484 ± 1.012 ^a^	1.699 ± 0.429 ^ab^	1.625 ± 0.065 ^bc^	0.273 ± 0.051 ^bc^	31.40 ± 5.44 ^ab^	79.23 ± 7.64 ^abc^	5.92 ± 0.93 ^abcde^	6.26 ± 1.23 ^b^
MA+AS	0.051 ± 0.005 ^a^	0.202 ± 0.012 ^ab^	0.391 ± 0.055 ^a^	0.020 ± 0.001 ^b^	0.773 ± 0.267 ^bc^	1.262 ± 0.170 ^abc^	1.533 ± 0.303 ^bc^	0.254 ± 0.042 ^bc^	30.71 ± 3.99 ^ab^	79.46 ± 5.20 ^abc^	5.96 ± 0.81 ^abcde^	5.97 ± 0.00 ^b^
MA+UR	0.054 ± 0.030 ^a^	0.212 ± 0.117 ^a^	0.460 ± 0.227 ^a^	0.019 ± 0.005 ^b^	1.395 ± 0.748 ^ab^	1.760 ± 0.324 ^ab^	2.916 ± 0.873 ^a^	0.357 ± 0.037 ^bc^	37.42 ± 19.82 ^a^	93.44 ± 51.30 ^a^	8.05 ± 3.45 ^abc^	6.56 ± 1.79 ^b^

**Table 5:** Treatments of sediment in the pot experiment.

Abbreviation	Treatment	Time of Application (Days after Sowing)
C	no treatment	-
AN_150_	2.14 g ammonium nitrate per pot (150 mg N/kg)	8 days
AN_300_	4.28 g ammonium nitrate per pot (300 mg N/kg)	8 days
AS_150_	3.54 g ammonium sulfate per pot (150 mg N/kg)	8 days
AS_300_	7.08 g ammonium sulfate per pot (300 mg N/kg)	8 days
UR_150_	1.61 g urea per pot (150 mg N/kg)	8 days
UR_300_	3.22 g urea per pot (300 mg N/kg)	8 days
OA	12.60 g oxalic acid dihydrate per pot (20 mmol/kg of oxalic acid)	35 days
MA	13.40 g malic acid per pot (20 mmol/kg of malic acid)	35 days
OA+AN	2.14 g ammonium nitrate per pot (150 mg N/kg)	8 days
	12.60 g oxalic acid dihydrate per pot (20 mmol/kg of oxalic acid)	35 days
OA+AS	3.54 g ammonium sulfate per pot (150 mg N/kg)	8 days
	12.60 g oxalic acid dihydrate per pot (20 mmol/kg of oxalic acid)	35 days
OA+UR	1.61 g urea per pot (150 mg N/kg)	8 days
	12.60 g oxalic acid dihydrate per pot (20 mmol/kg of oxalic acid)	35 days
MA+AN	2.14 g ammonium nitrate per pot (150 mg N/kg)	8 days
	13.40 g malic acid per pot (20 mmol/kg of malic acid)	35 days
MA+AS	3.54 g ammonium sulfate per pot (150 mg N/kg)	8 days
	13.40 g malic acid per pot (20 mmol/kg of malic acid)	35 days
MA+UR	1.61 g urea per pot (150 mg N/kg)	8 days
	13.40 g malic acid per pot (20 mmol/kg of malic acid)	35 days

## Data Availability

Data are contained within the article.
